# Human Bone Marrow-Derived Myeloid Dendritic Cells Show an Immature Transcriptional and Functional Profile Compared to Their Peripheral Blood Counterparts and Separate from Slan+ Non-Classical Monocytes

**DOI:** 10.3389/fimmu.2018.01619

**Published:** 2018-07-16

**Authors:** Nathalie van Leeuwen-Kerkhoff, Kristina Lundberg, Theresia M. Westers, Shahram Kordasti, Hetty J. Bontkes, Malin Lindstedt, Tanja D. de Gruijl, Arjan A. van de Loosdrecht

**Affiliations:** ^1^Cancer Center Amsterdam, Department of Hematology, VU University Medical Center, Amsterdam, Netherlands; ^2^Department of Immunotechnology, Lund University, Lund, Sweden; ^3^Department of Haematological Medicine, King’s College London, London, United Kingdom; ^4^Department of Pathology, VU University Medical Center, Amsterdam, Netherlands; ^5^Cancer Center Amsterdam, Department of Medical Oncology, VU University Medical Center, Amsterdam, Netherlands

**Keywords:** dendritic cells, non-classical monocytes, bone marrow, peripheral blood, microarray analysis, cytokines

## Abstract

The human bone marrow (BM) gives rise to all distinct blood cell lineages, including CD1c+ (cDC2) and CD141+ (cDC1) myeloid dendritic cells (DC) and monocytes. These cell subsets are also present in peripheral blood (PB) and lymphoid tissues. However, the difference between the BM and PB compartment in terms of differentiation state and immunological role of DC is not yet known. The BM may represent both a site for development as well as a possible effector site and so far, little is known in this light with respect to different DC subsets. Using genome-wide transcriptional profiling we found clear differences between the BM and PB compartment and a location-dependent clustering for cDC2 and cDC1 was demonstrated. DC subsets from BM clustered together and separate from the corresponding subsets from PB, which similarly formed a cluster. In BM, a common proliferating and immature differentiating state was observed for the two DC subsets, whereas DC from the PB showed a more immune-activated mature profile. In contrast, BM-derived slan+ non-classical monocytes were closely related to their PB counterparts and not to DC subsets, implying a homogenous prolife irrespective of anatomical localization. Additional functional tests confirmed these transcriptional findings. DC-like functions were prominently exhibited by PB DC. They surpassed BM DC in maturation capacity, cytokine production, and induction of CD4+ and CD8+ T cell proliferation. This first study on myeloid DC in healthy human BM offers new information on steady state DC biology and could potentially serve as a starting point for further research on these immune cells in healthy conditions as well as in diseases.

## Introduction

The human bone marrow (BM) compartment is the main site of hematopoiesis. It harbors early progenitor cells as well as more differentiated cells that will enter the circulation in a later stage for homing to secondary lymphoid organs or effector sites to mediate their immune functions. The developmental pathway of the dendritic cell (DC) lineage in the BM has been extensively studied in mice (1–12). Later, human homologs of DC were identified and recently the differentiation from human BM precursors into DC was described (13–23). Increasing knowledge on the sequential development of common and early progenitors to committed and more differentiated precursors may support the understanding of normal and aberrant hematopoiesis. This is for instance exemplified by selective cell lineage depletion in primary immunodeficiencies such as seen in patients with GATA2 mutations (24–26). These patients clearly show reduced numbers of natural killer cells, B cells, and monocytes and in addition a total absence of all DC subsets. In contrast, the level of granulocytes is normal, indicating a block in differentiation from progenitor to the more developed stages. Only recently, the immediate precursor of human DC has been identified which may now explain these DC-restricted depletions. Lee and colleagues developed a culture system in which they were able to define the human DC developmental pathway (18). From a common human granulocyte-monocyte-DC progenitor to a more committed human monocyte-DC progenitor, they finally could identify the direct human common DC progenitor (hCDP). The presence of hCDP is restricted to the BM compartment where it gives rise to a migratory conventional DC precursor (pre-CDC) that then produces the two major types of human myeloid DC, CD1c+, and CD141+ (now called cDC2 and cDC1, respectively) (19, 27). Transcriptional heterogeneity within the pre-CDC pool further revealed the existence of pre-commitment for cDC2 or cDC1 DC (20). Examination of different human tissues showed that these pre-CDC were not only present in the BM but could also be found in peripheral blood (PB) and tonsils. In contrast, the immediate monocyte precursor was only detected in BM. In addition to the information on sequential precursor production, data on activation state and functional capacities in different stages of cell development may give further insights into healthy and altered immune responses. It is assumed that immature/precursor forms of DC, including cDC2 and cDC1, leave the BM and circulate through the PB to the lymphoid tissues. Previously published data indeed support the fact that these circulating precursors are less mature and differentiated than their counterparts in peripheral tissues (15, 24, 28).

In human, the effect of environmental signals on DC function in different tissues, such as spleen, thymus, PB, lung, and skin was studied before by Heidkamp and colleagues (29).

However, information on developmental state and functional differences of cDC2 and cDC1 DC between the human BM and PB compartment is not available.

In this study, we focused on myeloid DC in the healthy human BM compartment in steady state conditions and compared them with their PB equivalents. Slan+ non-classical monocytes, previously recognized as a DC-like subset and, therefore, probably more closely linked to conventional DC than classical monocytes, were also included in this analysis. Detailed genome-wide transcriptional analysis as well as comparative functional validation showed clear compartment-related differences. Hierarchical clustering implied a close relationship of BM-derived cDC2 and cDC1 DC. Both subsets clustered away from their PB equivalents. Differentiation pathways, cell proliferation, and cell cycle control genes were most prominent in BM DC, whereas immune effector-related transcripts were over-expressed in PB subsets. In contrast, transcriptional profile and immunological function of slan+ non-classical monocytes were less dependent on the environment as a homogenous profile in both compartments was demonstrated. BM- and PB-derived slan+ non-classical monocytes clustered closer together than with the DC subsets from their respective compartments. Thus, under steady state circumstances both myeloid DC subsets potentially form a residential BM population that are in an earlier developmental phase than their PB equivalents, whereas slan+ non-classical monocytes are possibly PB-derived and patrol the BM in an effector role.

## Materials and Methods

### Sample Processing and Cell Isolation

Human BM was obtained after written informed consent from patients undergoing cardiac surgery in the VU University Medical Center Amsterdam, The Netherlands. Peripheral blood (buffy coat) was purchased from the Sanquin blood supply service (Amsterdam, The Netherlands). The study was approved by the local ethical committee and in accordance with the declaration of Helsinki. Mononuclear cells (MNCs) from both PB as well as from BM were isolated by density centrifugation using Ficoll-Paque medium (GE Healthcare, Uppsala, Sweden). For functional tests, conventional DC subsets (cDC2 and cDC1) were sorted from fresh samples with a BD FACSAria™ flow cytometer (BD Biosciences, San Jose, CA, USA), using a monoclonal antibody (mAb) cocktail consisting of CD1c-PE-Cy7 (eBioscience, San Diego, CA, USA), CD11c-PerCP-Cy5.5, CD14/CD19-APC-H7 (all, BD Biosciences), CD141-APC (Miltenyi Biotec, Utrecht, The Netherlands), and HLA-DR-V450 (BD Biosciences). Both cell subsets were identified in the CD11c+ and HLA-DR+ cell fraction. cDC2 cells were also gated as CD14−/CD19− (Figure S1A in Supplementary Material). For better recovery of viable cells, slan+ non-classical monocytes were isolated based on M-DC8 expression using a magnetic isolation kit (Miltenyi Biotec). For application in microarray experiments, all cell subsets were flowcytometrically sorted from four individual PB and four BM donors (non-paired samples). After an enrichment step, comprising magnetic depletion of CD3+ and CD19+ cells using microbeads (Miltenyi Biotec), cells were incubated with a mAb cocktail consisting of M-DC8-FITC (Miltenyi Biotec), CD16-PE (Beckman Coulter, Brea, USA), CD11c, CD1c, CD141, CD14, CD19, and CD45-KO (BD Biosciences, Figure S1B in Supplementary Material). Sorted cells routinely showed >92% purity and were stored in Trizol (Life Technologies, Carlsbad, CA, USA) until further use for RNA extraction.

### Cell Frequencies

**Figure 1:**
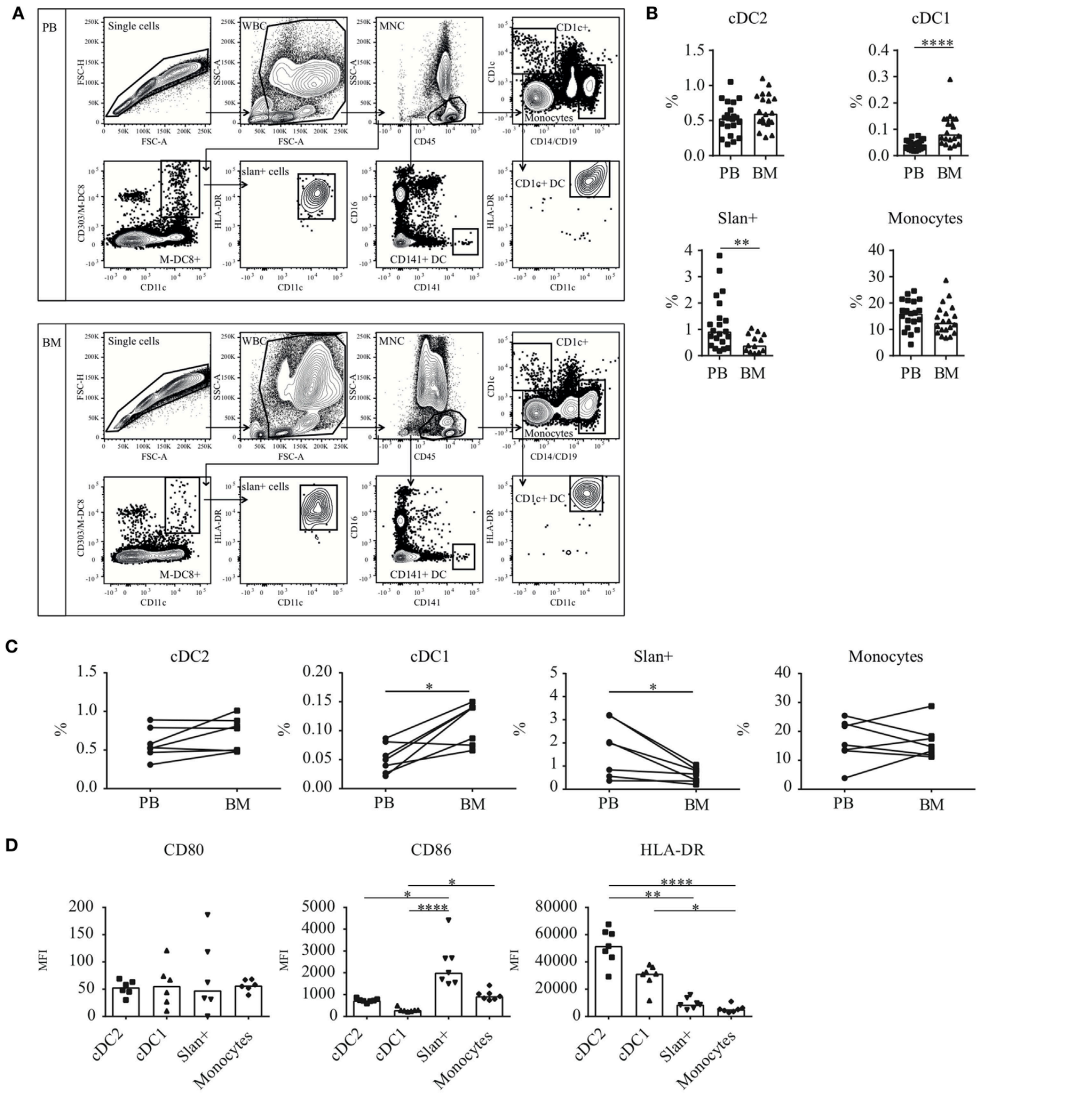

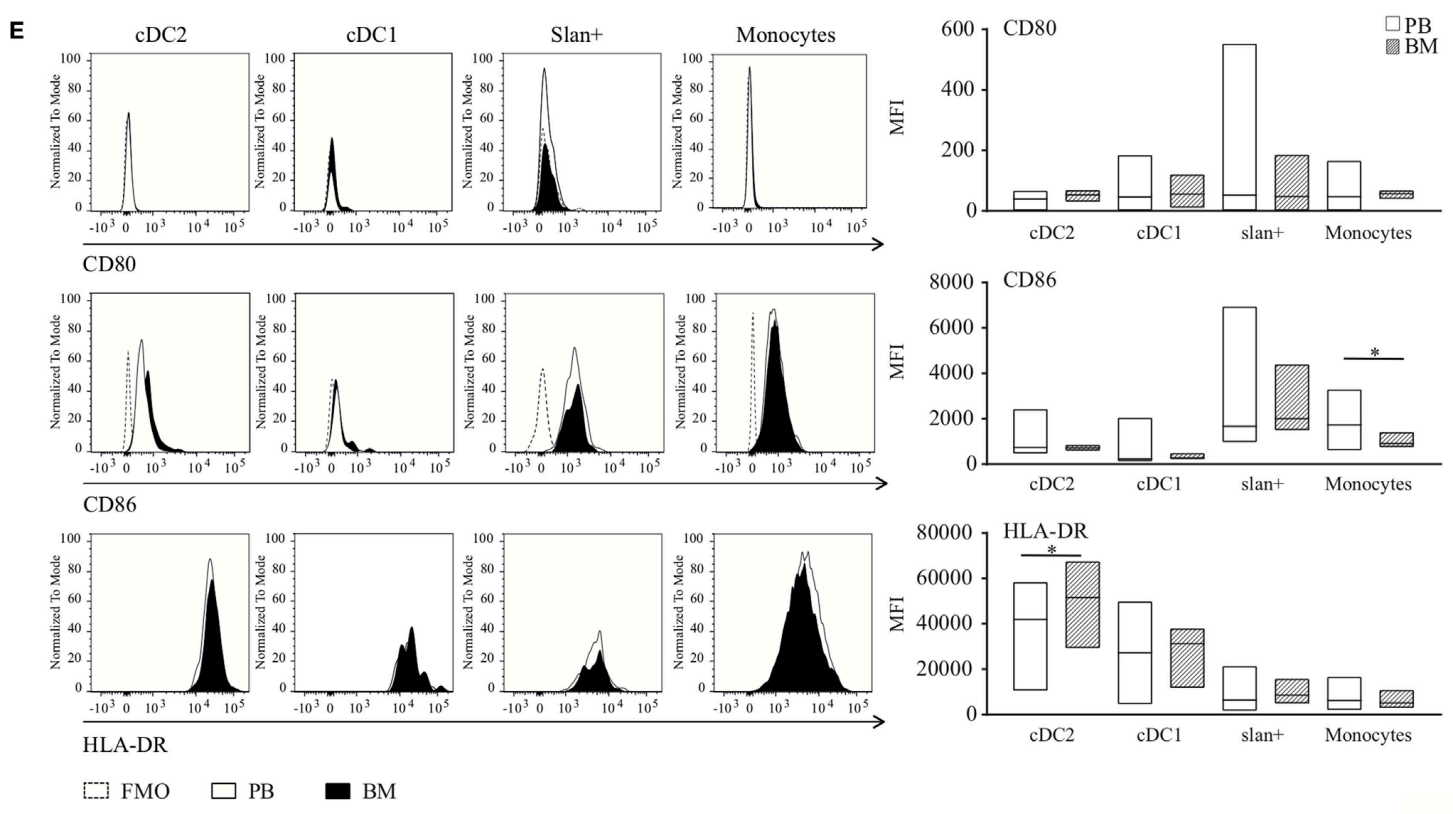
Dendritic cell (DC) and monocyte enumeration and baseline expression levels of (co-) stimulatory molecules in BM and PB. **(A)** Gating strategy for DC and monocyte subsets. CD45+ MNCs were gated after debris and doublet exclusion. Thereafter, cell subsets were identified based on the expression of CD1c (cDC2), CD141 (cDC1), or M-DC8 (slan+ non-classical monocytes). Classical monocytes were selected based on their high CD14 expression. **(B)** Frequencies of slan+ cells, monocytes, and the myeloid DC subsets cDC2 and cDC1 in human BM as compared to human PB. Percentages were calculated from the total CD45+ mononuclear cell fraction. Individual results and median percentages are shown for 17–21 (PB) and 13–20 (BM) healthy donors. Monocytes formed the main population in both compartments (PB, 16.0% and BM, 12.5%), followed by cDC2 (PB, 0.53% and BM, 0.60%) and slan+ non-classical monocytes (PB, 0.92% and BM, 0.39%). In BM as well as in PB, cDC1s were only present in low percentages (PB, 0.043% and BM, 0.081%). **(C)** Paired frequencies of PB and BM subsets, derived from one donor. A Wilcoxon matched-pairs signed rank test was performed to compare the PB and BM compartment (*n* = 7 donors, cDC2, and monocytes: no significant differences, cDC1 *p* = 0.03 and slan+ *p* = 0.02). **(D)** Baseline expression levels of HLA-DR (*n* = 7) and the co-stimulatory molecules CD80 (*n* = 6) and CD86 (*n* = 7) on BM-derived cell subsets. Individual experiments and median values are shown. **(E)** A comparison of baseline expression levels of three different activation markers between cell subsets from the PB (*n* = 14–23) and BM (*n* = 6–7) compartment. Histograms of the different markers of a representative donor and bar graphs of repeated experiments are shown. Median MFI values of individual stainings of the different cell subsets are compared between the two compartments. For CD80 and CD86, FMO controls are shown as a dotted line. Abbreviations: BM, bone marrow; PB, peripheral blood; WBC, white blood cells; MNC, mononuclear cells; MFI, median fluorescence intensity; FMO, fluorescence minus one. *p < 0.05, **p < 0.01, ***p < 0.001, and ****p < 0.0001.

The enumeration of different cell subtypes in BM and PB was performed after lysis of erythrocytes (BD Pharm Lyse, BD Biosciences). Samples were incubated with a mix of mAb: M-DC8-FITC, CD303-FITC (Miltenyi Biotec), CD16-PE, CD11c-PerCP-Cy5.5, CD1c-Pe-Cy7, CD141-APC, CD14-APC-H7, CD19-APC-H7, HLA-DR-V450, and CD45-KO and analyzed on a flow cytometer (FACSCanto-II, BD Biosciences). Data analysis was done using FlowJo software (Tree star, Ashland, OR, USA). After debris and doublet exclusion, myeloid cell subsets were identified in the CD45+ compartment and further gated on CD1c, CD141, M-DC8, CD303, or CD14 expression. HLA-DR and CD11c were used for further selection of cell populations (Figure 1A). To exclude contamination of B cells in the CD14+ monocyte gate, back gating in CD45 vs side scatter plots was used (data not shown in figure). Frequencies were calculated as percentages of CD45+ MNCs.

### Preparation of cRNA and Gene Chip Hybridization

RNA was isolated from cDC2 (18,000–265,000 cells), cDC1 (1,000–17,300) and slan+ non-classical monocytes (9,000–150,000) and amplified using the Ovation Pico WTA System V2 (NuGen, San Carlos, CA, USA). Subsequently, RNA was labeled with the Encore Biotin Module Kit (NuGEN) according to manufacturer’s protocols. Five microgram of the resulting labeled cDNA from each sample was hybridized to Human Transcriptome Arrays 2.0 microarrays (Affymetrix) and signals were scanned by Affymetrix GeneChip Scanner 3000 7G.

### Microarray Data Analysis

Data analysis was performed as previously described (30). Briefly, microarray signals were normalized using the RMA algorithm in the Affymetrix^®^ Expression Console software (Affymetrix^®^) and quality checks and background noise correction were performed. Qlucore Omics Explorer 3.2 (Qlucore AB, Lund, Sweden) and Perseus were used for transcript filtering, identification of differentially expressed genes (DEGs; by performing an ANOVA analysis and using *q*-values of <0.05), heat map visualization, and Gene Set Enrichment Analysis (GSEA). Pathway analyses and interaction networks were created within the STRING v10.0 database (http://string-db.org/). The microarray data have been deposited in the GEO public database under Accession Number GSE112770.

### Cell Staining, Maturation, and Cytokine Secretion

The intracellular expression of the proliferation marker Ki-67 was assessed in different cell subsets of the myeloid lineage by using a mAb panel consisting of the surface markers CD1c, CD11c, CD14, CD19, CD45, CD141, M-DC8, and HLA-DR, and, a PE-conjugated mAb against the intracellular Ki-67 marker (BD Pharmingen). Unstimulated cell subsets of eight paired BM and PB samples were analyzed. After erythrocyte lysis, cell surface staining was performed. Thereafter, cells were fixed with phosphate-buffered saline containing 1% paraformaldehyde (PFA) and permeabilized by means of BD FACS Lysing solution (BD FACS Lysing Solution, BD Biosciences). Subsequently, the Ki-67 mAb was added and its intracellular expression in the different cell subsets was evaluated by flow cytometry (FACSCanto-II, BD Biosciences).

Baseline expression levels of co-stimulatory (CD80 and CD86) and minor histocompatibility (MHC)-class II (HLA-DR) molecules were evaluated before isolation of BM and PB cell subsets. Assessment of maturation capacity upon TLR-ligation by DC subsets and slan+ non-classical monocytes from both compartments was performed as described previously (30). Briefly, cells were isolated and pre-incubated for 4 h in Iscove’s Modified Dulbecco’s Medium supplemented with 10% fetal calf serum, 100 IU/ml penicillin, and 100 µg/ml streptomycin (all Gibco, Paisley, United Kingdom) at 37°C in a 5% CO_2_ incubator. Thereafter, they were left unstimulated or were stimulated overnight with a combination of TLR ligands, i.e., LPS (100 ng/ml, Sigma-Aldrich, St. Louis, MO, USA) and R848 (3 µg/ml, Enzo Life Sciences, Farmingdale, USA) for cDC2 and slan+ non-classical monocytes, or Poly I:C (25 µg/ml, Sigma-Aldrich) and R848 for cDC1. The expression of respective markers was again measured by flow cytometry. Supernatants of these cultures were used for the detection of different cytokines by human enhanced sensitivity cytometric bead array (CBA) flex sets (BD Biosciences).

### Allogeneic Mixed Leukocyte Reaction (MLR)

The capacity of DC subsets and slan+ non-classical monocytes to induce T cell proliferation was tested in an allogeneic MLR as described previously (30, 31). In brief, carboxyfluorescein succinimidyl ester (CFSE, Life Technologies, 1 μM)-labeled PB lymphocytes (PBL, i.e., CD14 depleted cell fraction) were co-cultured for 5 days with isolated and stimulated BM- or PB-derived slan+ monocytes or cDC2 in a ratio of 1:10. CFSE-dilution in CD4+ and CD8+ T cells was measured by flow cytometry.

### Statistical Analysis

Graphpad Prism 6 software (San Diego, CA, USA) was used for non-microarray statistical data analysis. For two-group comparisons a non-parametric Mann–Whitney *U* test was performed, whereas for matched sample analyses a Wilcoxon signed rank test was used. For statistical differences a *p*-value of <0.05 was considered significant.

## Results

### Quantitative Differences in Subsets Between the BM and PB

Dendritic cell and monocyte subsets were enumerated in the mononuclear cell fraction of fresh PB (*n* = 17–21) and BM (*n* = 13–20) samples from non-paired healthy donors (HD). Cell populations were characterized by the expression of the cell-identifying markers CD1c and CD141 for the myeloid DC subsets. cDC2 were further defined as CD19−/CD14− and HLA-DR+/CD11c+. Slan+ cells, recently identified as the non-classical monocytes (30, 32), were characterized based on their CD11c and M-DC8 expression. Backgating in CD16 confirmed the expression of this marker on their cell surface (not shown). In contrast, classical monocytes did not express CD16, and were identified based on the high expression of CD14 (Figure 1A). B cell contamination was ruled out by creating back gating plots using discriminative CD45 expression and side scatter position of monocytes (not shown). In both compartments, all cell subsets could be found. Among these cells, CD14+classical monocytes accounted for the main population in PB as well as in BM (Figure 1B; PB, 16.0%; BM, 12.5%). Slan+ non-classical monocytes showed elevated numbers in PB compared to BM (PB, 0.92%. BM, 0.39%, *p* = 0.0095). In contrast, for cDC1, the frequency was significantly increased in the BM compared to PB (PB, 0.043%. BM, 0.081%, *p* < 0.0001). Comparable frequencies were detected for BM- and PB-derived cDC2 (PB, 0.53%; BM, 0.60%). In a set of 7 paired frequency analyses (PB and BM derived from the same donors), similar results were found for the different cell populations as compared to the larger unpaired cohort. For cDC1, increased percentages were seen in BM, whereas for slan+ non-classical monocytes all one-to-one comparisons showed elevated numbers in the PB compared to the BM (Figure 1C).

Extensive immunophenotypic information for DC and monocyte subsets in PB is available from previously published research (27, 30, 33–38). However, for human BM DC and monocytes these data are very limited (29). Therefore, the expression levels of important co-stimulatory and antigen-presenting molecules were assessed in BM-derived cells and compared to the expression levels on their PB counterparts. Similar to our observations for PB cell subsets (30), CD86 and HLA-DR were clearly expressed (at baseline, i.e., unstimulated expression) on DC and monocyte subsets. The expression of CD86 was higher on BM-derived monocyte subsets compared to BM-derived DC, whereas HLA-DR showed elevated levels on DC subsets (Figure 1D). Baseline expression levels of these markers were minimally different between BM and PB (Figure 1E). Only the HLA-DR expression on BM cDC2 was significantly higher compared to PB cDC2. For monocytes, CD86 levels were significantly higher in PB.

### Transcriptomic-Based Clustering of DC Subsets but Not of Slan+ Non-Classical Monocytes Largely Depends on the Compartment They Reside in

**Figure 2:**
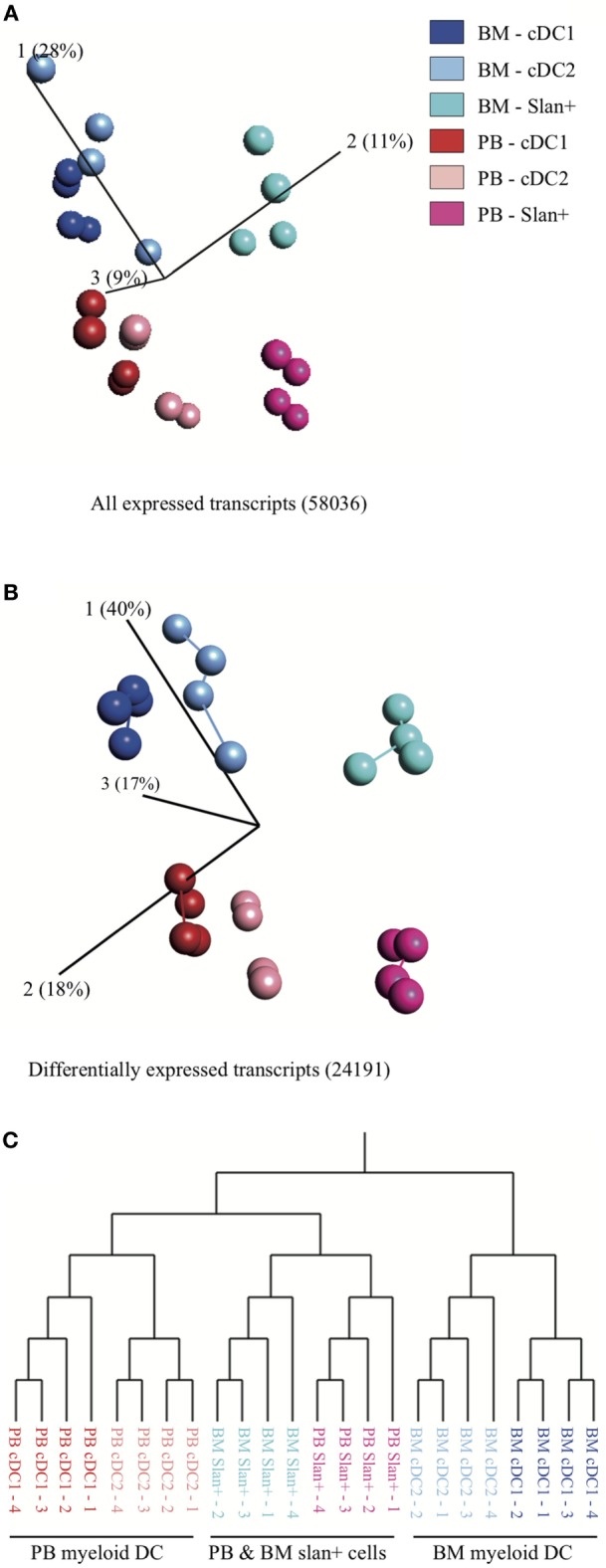
Principal component analysis (PCA) and hierarchical clustering of cell subsets in bone marrow (BM) and peripheral blood (PB). Relationship between slan+ monocytes and cDC2 and cDC1 (myeloid DC) in the two different compartments: PB vs BM. For each subset, isolated cells of four PB and four BM donors were used. **(A)** PCA for all expressed transcripts and **(B)** PCA for genes that were differentially expressed between the cell subsets. The length of the lines between the dots corresponds to the degree of similarity (minimal spanning tree analysis). Percentages shown indicate the degree of total variation within each component. **(C)** Hierarchical clustering, based on differentially expressed transcripts, of replicate samples.

For a better insight into the function of myeloid DC subsets (cDC2 and cDC1) in the BM compartment and to potentially identify compartment-related transcriptomic differences, an extensive genome-wide profiling study was performed. Both DC subsets as well as slan+ non-classical monocytes were sorted from fresh PB and BM of four individual HD. A total of 58,036 transcripts were found to be expressed. Principal component analysis (PCA), using all expressed transcripts, showed a strong similarity of cell subsets between donors (Figure 2A). After applying an ANOVA test, 24,191 genes were found to be differentially expressed between all subsets in both compartments. Minimal spanning tree analysis revealed a compartment-based separation of cDC2 and cDC1. In contrast, slan+ non-classical monocytes from both compartments clustered closer together and separated from the DC subsets, irrespective of compartment (Figure 2B). Hierarchical clustering confirmed the separation for the conventional cDC2 and cDC1 subsets. Slan+ non-classical monocytes from PB and from BM grouped together (Figure 2C). Furthermore, slan+ non-classical monocytes were more closely related to PB DC than to BM DC subsets. The same analysis was repeated for DC only, without using slan+ monocytes. PCA and hierarchical clustering of all genes and 10114 DEGs showed similar results (Figure S2 in Supplementary Material). These findings suggest that transcriptional expression in myeloid DC subsets is more influenced by their environment (BM or PB) than by their intrinsic subset characteristics. In contrast, gene expression in slan+ non-classical monocytes appeared to be less dependent on environmental factors.

### DC Subsets Display a Less Immune-Active Transcriptional Signature Than Slan+ Non-Classical Monocytes in the BM

**Figure 3:**
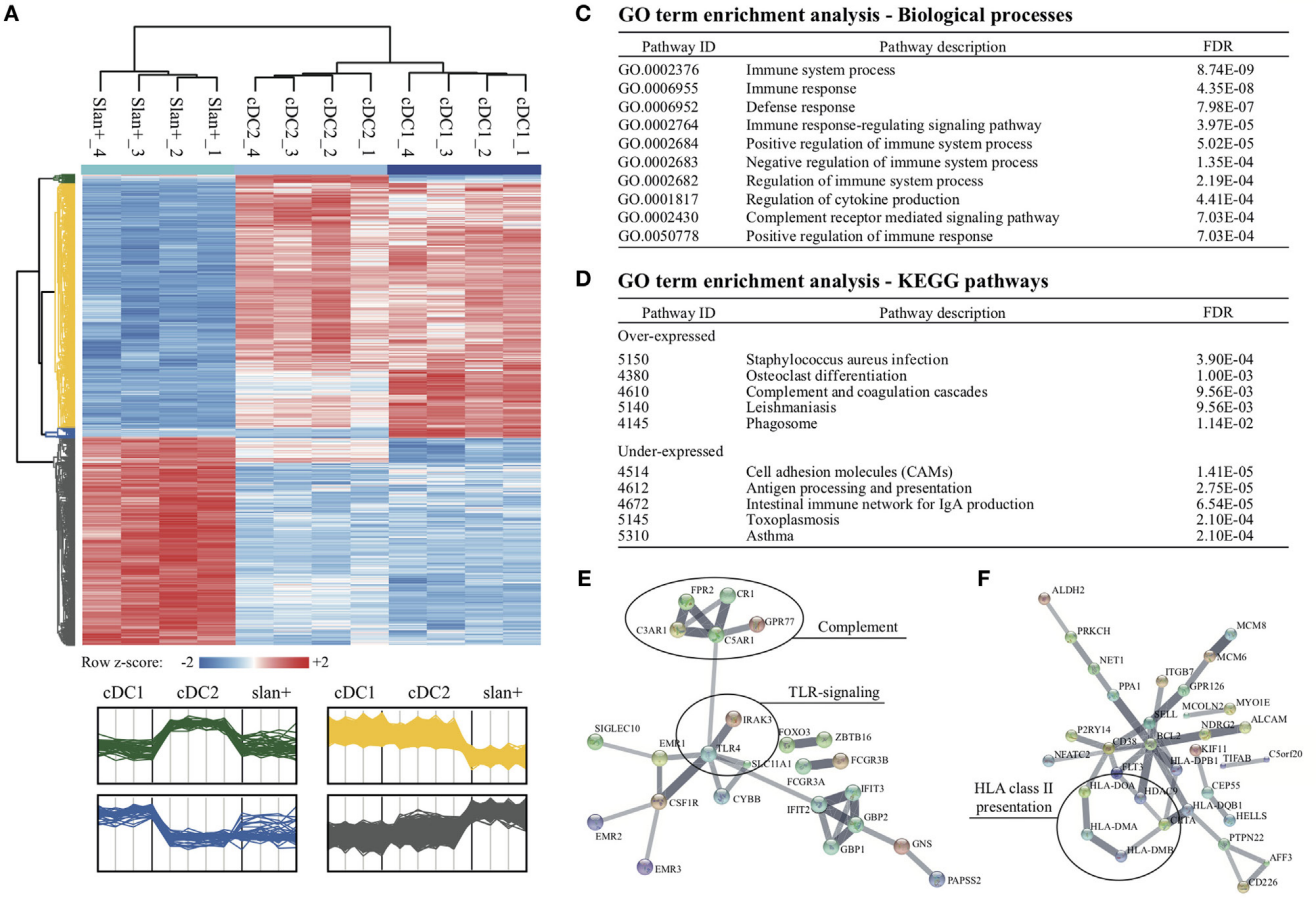

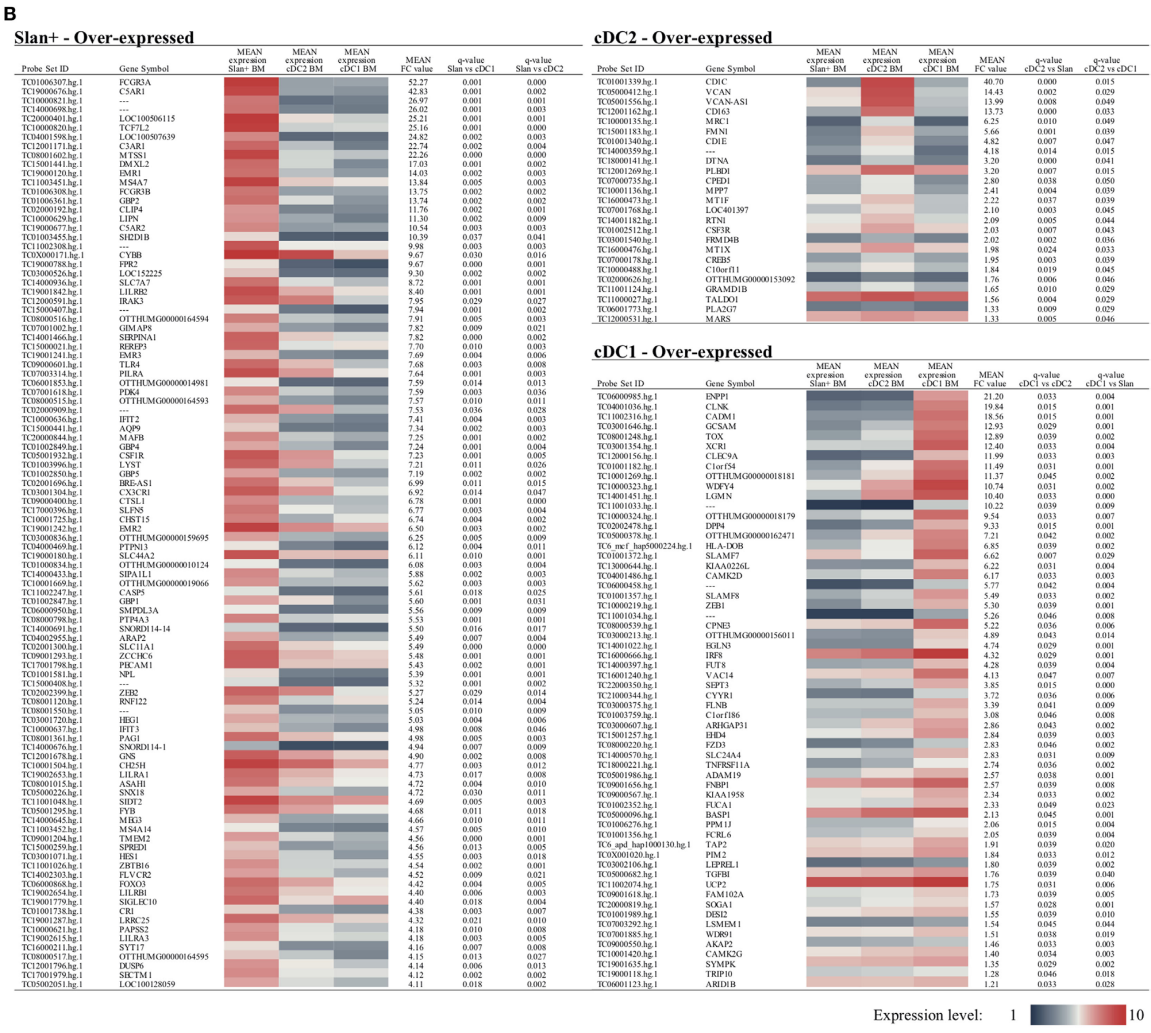
Differentially expressed genes (DEGs) between slan+ monocytes and cDC2 and cDC1 in the bone marrow (BM) compartment. After identification, DEGs were further analyzed for the three cell subsets in the BM compartment. **(A)** Cluster analysis of transcripts that are differentially expressed between the subsets are presented in a heat map. Each row represents a single transcript. Relative expression levels for four donors per subset are shown. *z*-scores were calculated in one row to show the relative expression values per donor compared to the mean of the entire row (*z*-score −2: low expression, *z*-score +2: high expression). Four different clusters were generated. In green, DEGs that were over-expressed in cDC2 are shown, whereas DEGs over-expressed in cDC1 are within the blue cluster. Under- and over-expressed transcripts for slan+ monocytes are shown in yellow and gray, respectively. **(B)** Coding and over-expressed transcripts, sorted on mean FC values, are shown for all cell subsets. For slan+ monocytes the top 100 is used, whereas for the other two cell subsets 25 and 60 transcripts are shown (cDC2 and cDC1, respectively). For each transcript, the mean absolute expression level of four donors (1: low expression, 10: high expression) is color coded in the given cell subset. Q-values for both comparisons (i.e., slan+ vs cDC2 and slan+ vs cDC1) are shown. **(C)** Pathway analysis of over-expressed transcripts in slan+ monocytes compared to both DC subsets. The top 100 highest FC values from over-expressed coding transcripts were imported into the STRING v10.0 bioinformatics tool and significant enriched pathways were identified. From this list, the top 10 with lowest FDR (out of 62) of enriched biological processes are shown. **(D)** Enriched KEGG pathways for over- and under-expressed transcripts in slan+ monocytes compared to DC subsets. Top 100 highest FC values of coding transcripts were used from the over- and under-expressed gene lists. An interaction network analysis of involved transcripts is displayed for over-expressed genes **(E)** as well as for under-expressed genes **(F)** in slan+ monocytes. Disconnected nodes are hidden and the line thickness indicates the strength of data support. Abbreviations: FC, fold change; FDR, false discovery rate; GO, gene ontology; ID, identification; KEGG, Kyoto encyclopedia of genes and genomes.

Recent gene expression profile studies mainly focused on PB-derived cell types. For human BM-derived monocytes and DC, limited, if any, transcriptional information is available. Therefore, detailed gene expression profiles were analyzed in BM-derived slan+ non-classical monocytes, cDC2, and cDC1. From all expressed BM genes (58,459), differentially expressed transcripts between the three subsets were listed. Only if a gene was differentially over- or under-expressed in both comparative analyses (e.g., slan+ vs cDC2 and slan+ vs cDC1), it was considered to be subset-specific. Similar to our previous findings for PB subsets (30), BM-derived slan+ non-classical monocytes showed the highest number of unique transcripts. In total, 2,142 genes were differentially expressed in slan+ non-classical monocytes as compared to both DC subsets. From these DEGs, 965 were over-expressed and 1,177 were under-expressed. cDC2 showed 31 over-expressed and 14 under-expressed transcripts compared to the other two subsets. For cDC1 69 genes were found to be over-expressed and 54 to be under-expressed. Clustering of these DEGs in a heat map identified four gene clusters (Figure 3A, lower panel). Absolute expression levels of the identified DEGs offered further details of the possible biologically relevant differences between the subsets (Figure 3B). Consistent with our PB data (30), the top 100 over-expressed genes for slan+ non-classical monocytes contained recognized monocytic and immune-related genes, such as *FCGR3A/B, TLR4, C5AR1/2, C3AR1, CR1*, and *CX3CR1*. For cDC2, 25 genes with highest FC values are shown and for cDC1 60 genes. Also for the two BM-derived DC subsets well-known transcripts were found (*CD1c, CD163, MRC1*, and *CSF3R* for CD1c DC and *XCR1, CLEC9A*, and *IRF8* for CD141 DC), indicating a consistent expression of subset-defining genes in BM and PB. Due to the limited number of over-expressed transcripts in cDC2 and cDC1, further pathway and interaction analyses were only performed for slan+ non-classical monocytes in order to find relevant biological context for these DEGs. Again, the top 100 highest FC values were used in this approach. They were imported in the STRING v10.0 database and a gene ontology (GO) term enrichment analysis was executed. The most enriched pathways mainly covered immune-related biological processes. As found for PB-derived slan+ non-classical monocytes (30), their BM counterparts also appeared to be highly involved in complement-mediated immune responses (Figures 3C,D). Enrichment analysis for under-expressed DEGs pointed to a lower involvement in adhesion and antigen processing and presentation (Figure 3D). Yet again, this shows great overlap with previous PB findings (30). Network interaction analysis of over-expressed DEGs showed a strong interaction of complement-related proteins as well as TLR-mediated signaling pathways (Figure 3E). For under-expressed DEGs a strong network of MHC-class II molecules was found (Figure 3F).

### Gene Expression Profiles Are Dependent on Their Environment

**Figure 4:**
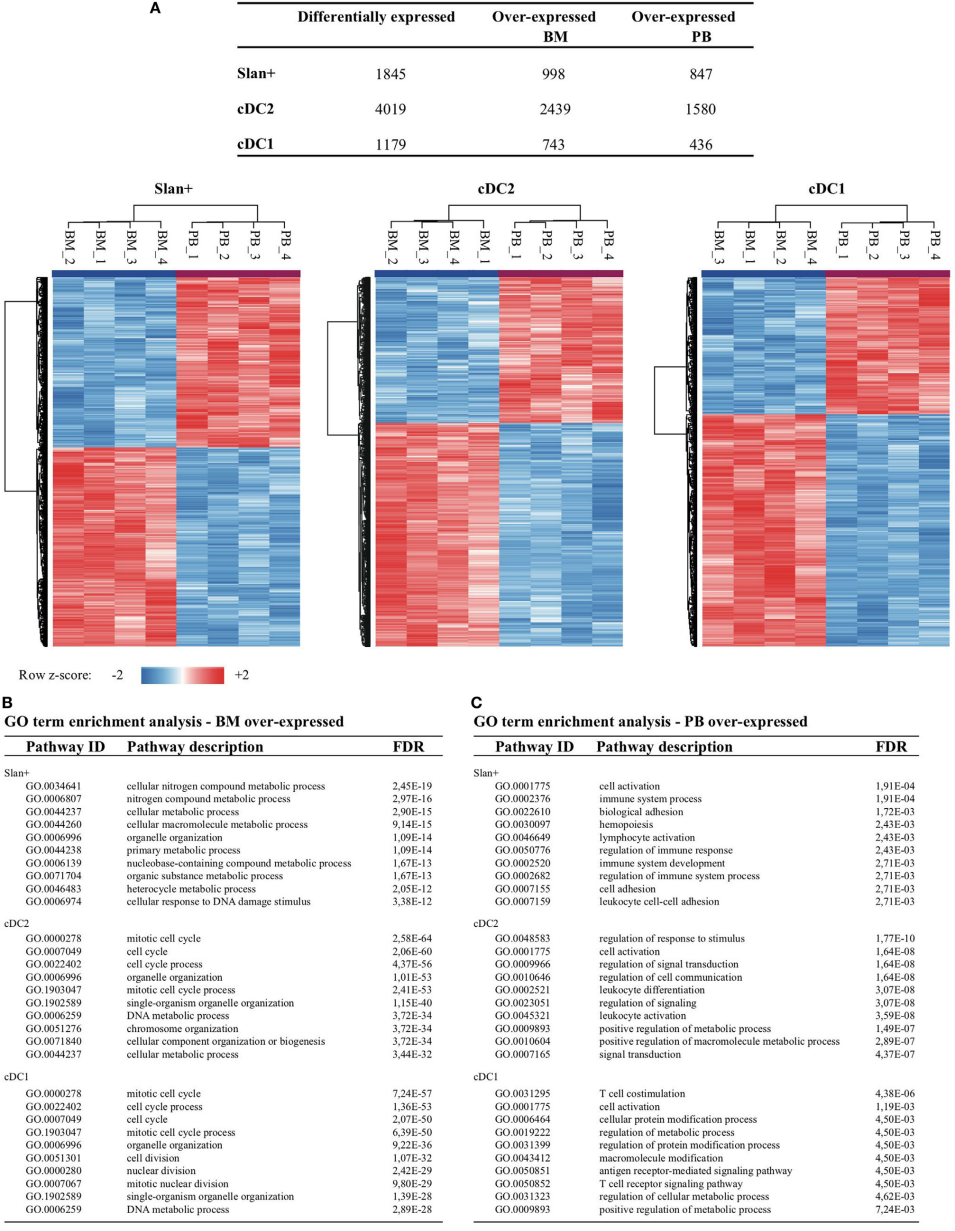

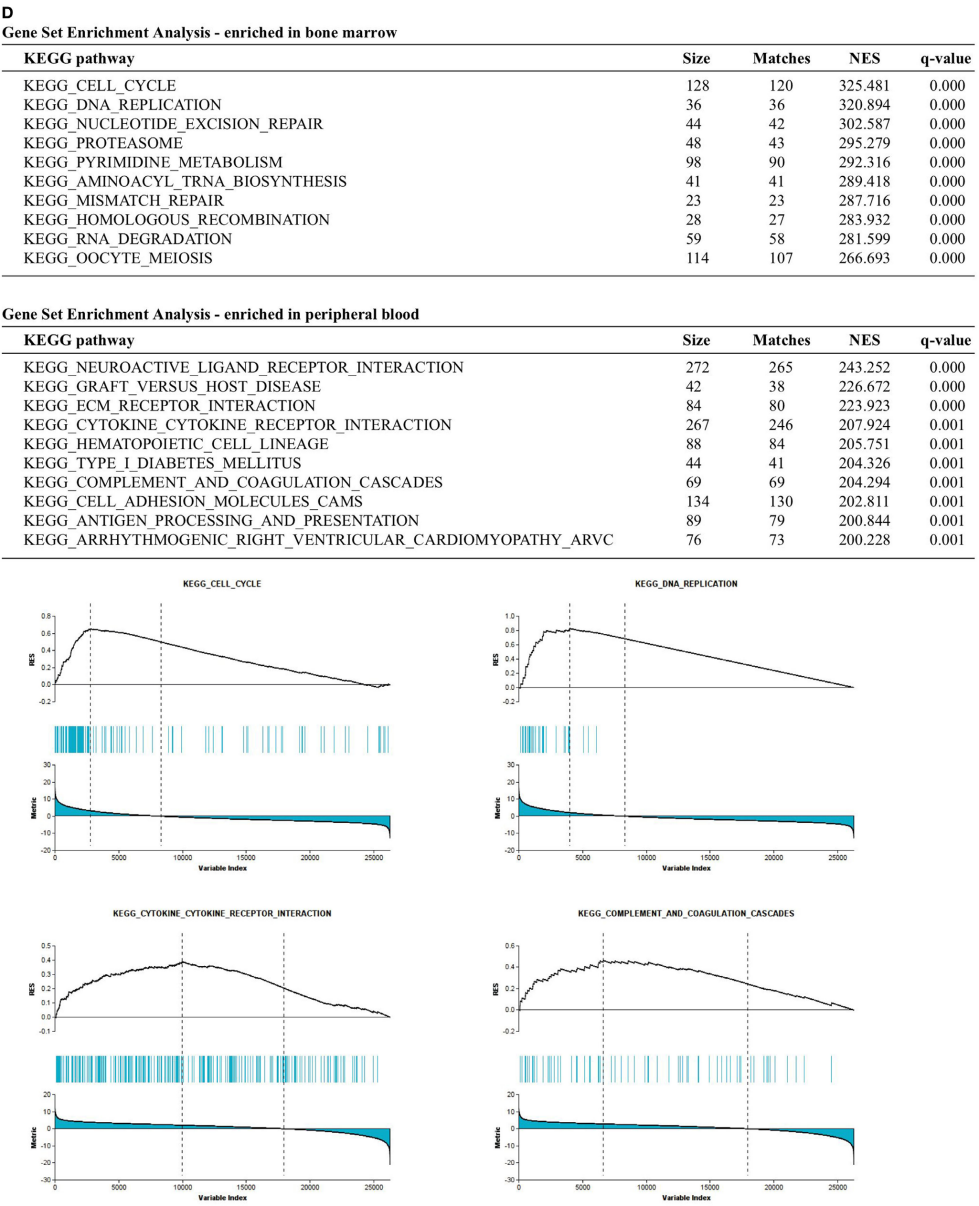
Transcriptomic comparison between cell subsets of the bone marrow (BM) vs the peripheral blood (PB) compartment. Gene expression profiles were compared between the BM and PB for each individual cell subset. **(A)** Number of differentially expressed transcripts found between the two different compartments (e.g., slan+ in BM vs slan+ in PB). Differentially expressed genes (DEGs) were clustered per row by using z-scores for each transcript, thereby reflecting relative expression levels. Heat maps for each individual subset are shown. For each cell subset, a pathway enrichment analysis was performed in which all DEGs were used that were over-expressed in BM **(B)** or over-expressed in PB **(C)**. Top 10 enriched biological processes for both compartments and each individual subset are shown. **(D)** Gene set enrichment analysis for BM- and PB-derived cell subsets. The top 10 of enriched KEGG pathways are shown together with four selected enrichment plots. Abbreviations: FDR, false discovery rate; GO, gene ontology; ID, identification; NES, normalized enrichment score.

To further delineate the distinct gene expression profiles for cDC2 and cDC1 in relation to their compartment (and in a lesser extent for slan+ monocytes), DEGs between PB and BM were identified for all three individual subsets. The highest number of differentially expressed transcripts between BM and PB was found for cDC2: in total 4,019 DEGs, of which 2,439 DEGs were over-expressed in BM and 1,580 over-expressed in PB. For cDC1 743 genes were over-expressed in BM and 436 in PB. Slan+ non-classical monocytes showed 998 genes to be over-expressed in BM and 847 in PB. Clustering of relative expression levels in each subset is depicted in Figure 4A. In order to translate these identified DEGs into a relevant biological meaning, lists of over- and under-expressed genes were loaded onto the STRING v10.0 pathway tool. For all three subsets, but most distinctly for cDC2 and cDC1, cell cycle and division processes were highly enriched in BM-derived subsets. Also metabolic cellular responses were significantly overrepresented in BM-derived cells (Figure 4B). Genes that were over-expressed in PB subsets were mainly involved in immune-related pathways, indicating a more differentiated and immunologically functional profile for cells circulating in this compartment (Figure 4C). To further confirm these findings a GSEA was performed for BM as well as for PB subsets. Using Kyoto Encyclopedia of Genes and Genomes pathways from the Broad Institute database an enrichment of pathways involved in cell cycle and replication was found for BM subsets. In contrast, immune-related pathways were enriched in PB-derived subsets (Figure 4D). Again, confirming a distinct transcriptional profile of cell subsets in BM and PB.

### Generation of a Gene Signature for DC Subsets That Is Environmentally Dependent

**Figure 5:**
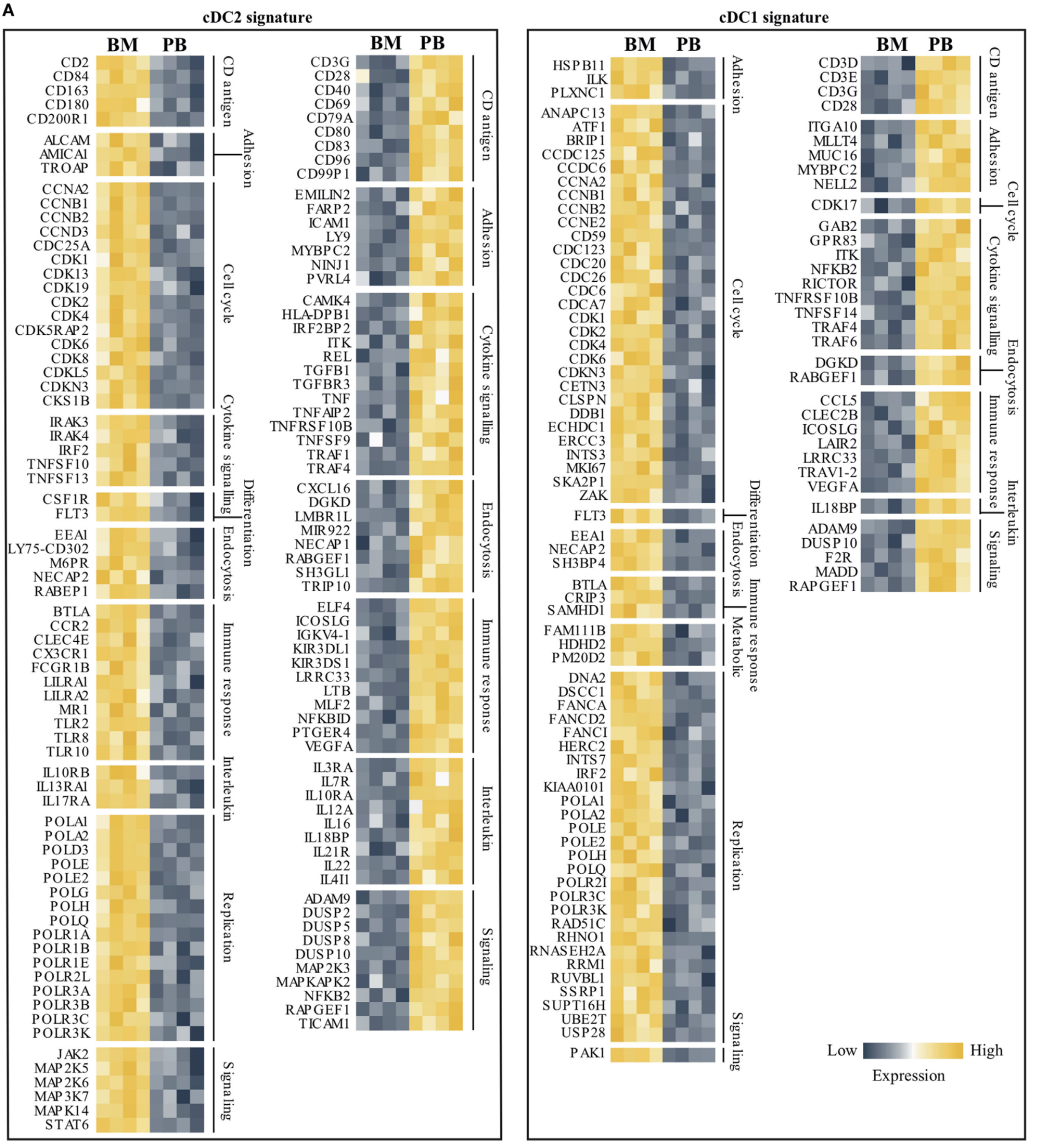

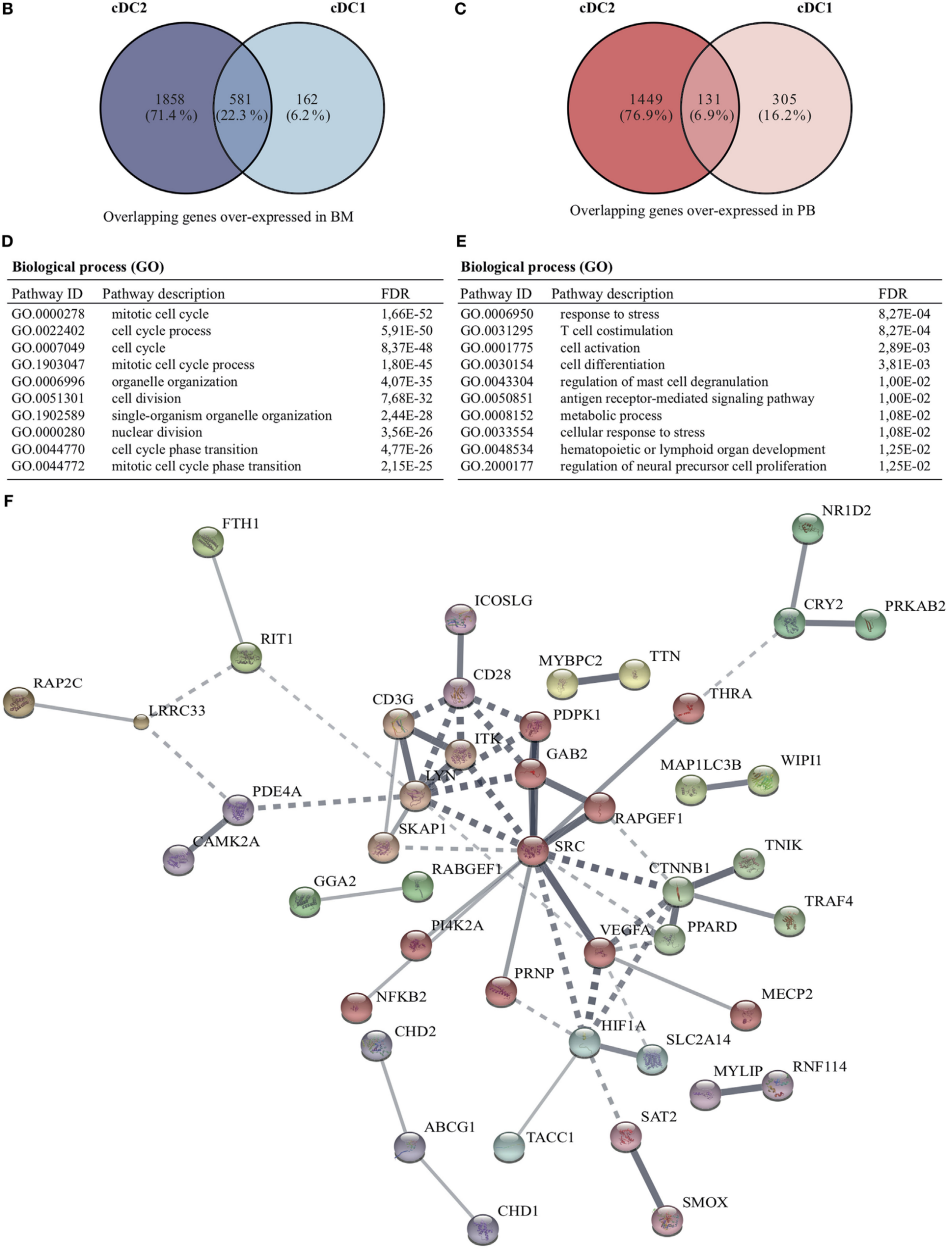
Compartment-specific DC gene signatures for cDC2 and cDC1. Both myeloid DC subsets were further analyzed to find compartment-specific DC gene signatures. **(A)** Transcripts were filtered based on gene descriptions and GO biological process terms by using cell cycle- and immune response-related terms. Relative expression for cDC2 and cDC1 in both bone marrow (BM) as well as peripheral blood (PB) are shown for selected genes. Venn diagrams were used to obtain overlapping genes between cDC2 and cDC1 in the BM **(B)** and PB **(C)** compartment. **(D)** The 581 commonly over-expressed transcripts in BM-derived DCs were used in a pathway enrichment analysis. **(E)** Similarly, overlapping transcripts (131 in total) of PB cDC2 and cDC21 were used in the STRING pathway analysis. **(F)** An interaction network of the PB transcripts was generated. In total, 85 genes could be annotated. The minimum required interaction score was set to medium confidence (0.400), disconnected nodes were hidden and the thickness of the lines indicates the strength of data support. Abbreviations: FDR, false discovery rate; GO, gene ontology; ID, identification.

To establish a DC signature for BM as well as for PB, over-expressed gene lists (from the comparison BM vs PB) from both myeloid DC subsets were used. Since slan+ non-classical monocytes clearly form a non-DC-like population, they were excluded from this analysis. Using gene descriptions and GO biological process terms (e.g., cell cycle and immune response), transcripts involved in cellular and immunological processes were first selected for cDC2 as well as for cDC1. Relative expression levels of these transcripts are shown for BM and PB for both DC subsets (Figure 5A). From this selection, it is clear that transcripts that are involved in DNA replication, differentiation (i.e., *FLT3*), and cell cycle processes were evidently over-expressed in BM DC compared to PB DC. In contrast, cytokine signaling, adhesion, and endocytosis are terms that were overrepresented in PB DC. For PB cDC2, also co-stimulatory molecules such as CD40, CD80, and CD83 were expressed in higher levels compared to their BM equivalents. To further search for biologically relevant, and compartment-specific, DC gene signatures, Venn diagrams were created using gene lists of BM DC and PB DC. This revealed an overlap in over-expressed genes of 581 (22.3%) between BM cDC2 and BM cDC1 and 131 (6.9%) between PB cDC2 and PB cDC1 (Figures 5B,C), indicating a greater similarity between BM DC than between PB DC. Next, both overlapping genes lists were used for further pathway and network analysis in STRING v10.0. Pathways involved in cell cycle processes and cell division represented the overlapping transcripts in BM DC and formed the main biological function of these DC in this compartment (Figure 5D). In contrast, the overlapping transcripts in PB-derived DC could be found in pathways involving cell activation, differentiation, and antigen-receptor mediated signaling (Figure 5E). A network of these common PB-derived DC transcripts was generated (Figure 5F). Since the number of overlapping transcripts in BM DC was too high, a network could not be created.

### Functional Analyses Confirm Transcriptional Findings

**Figure 6:**
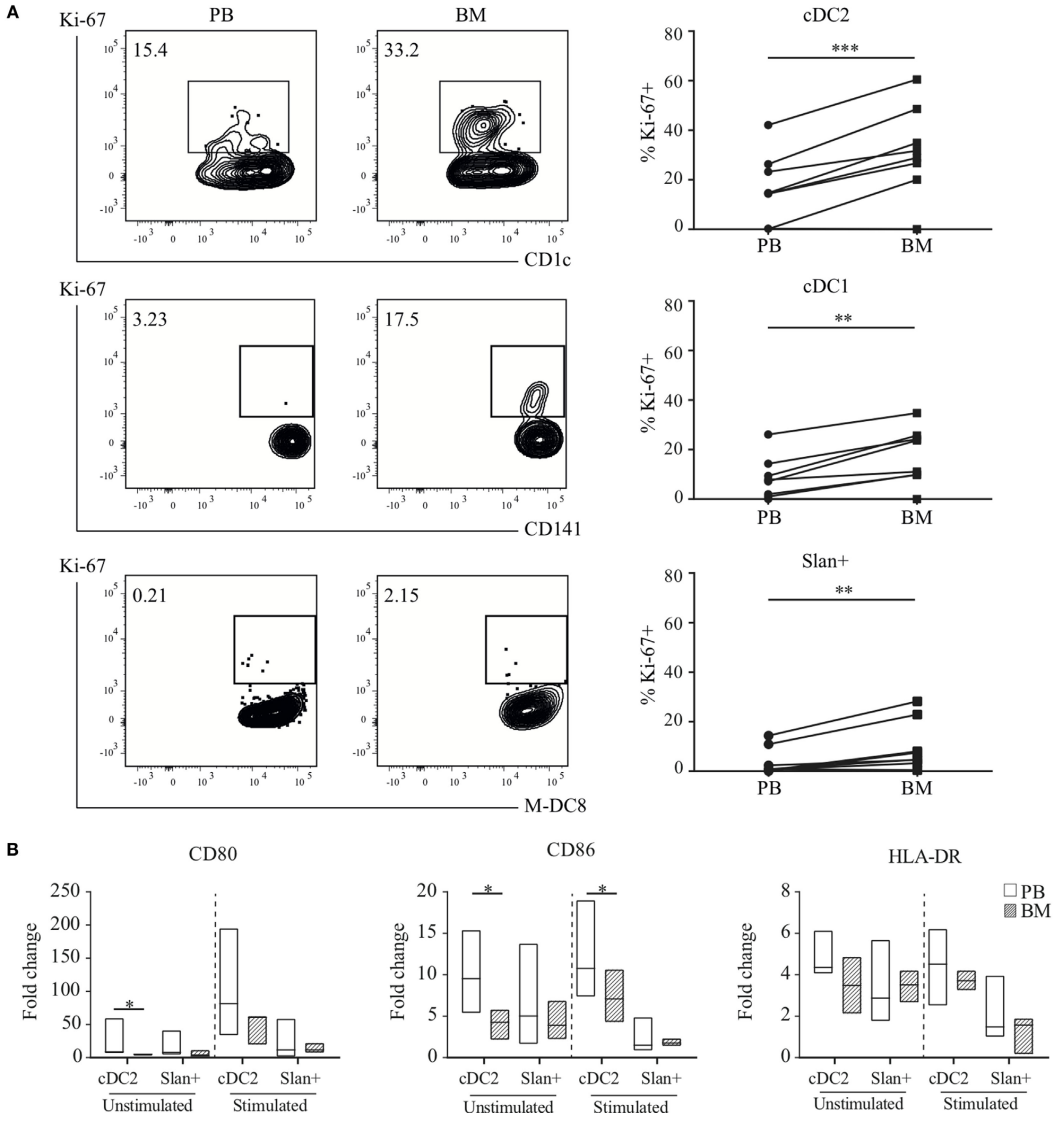

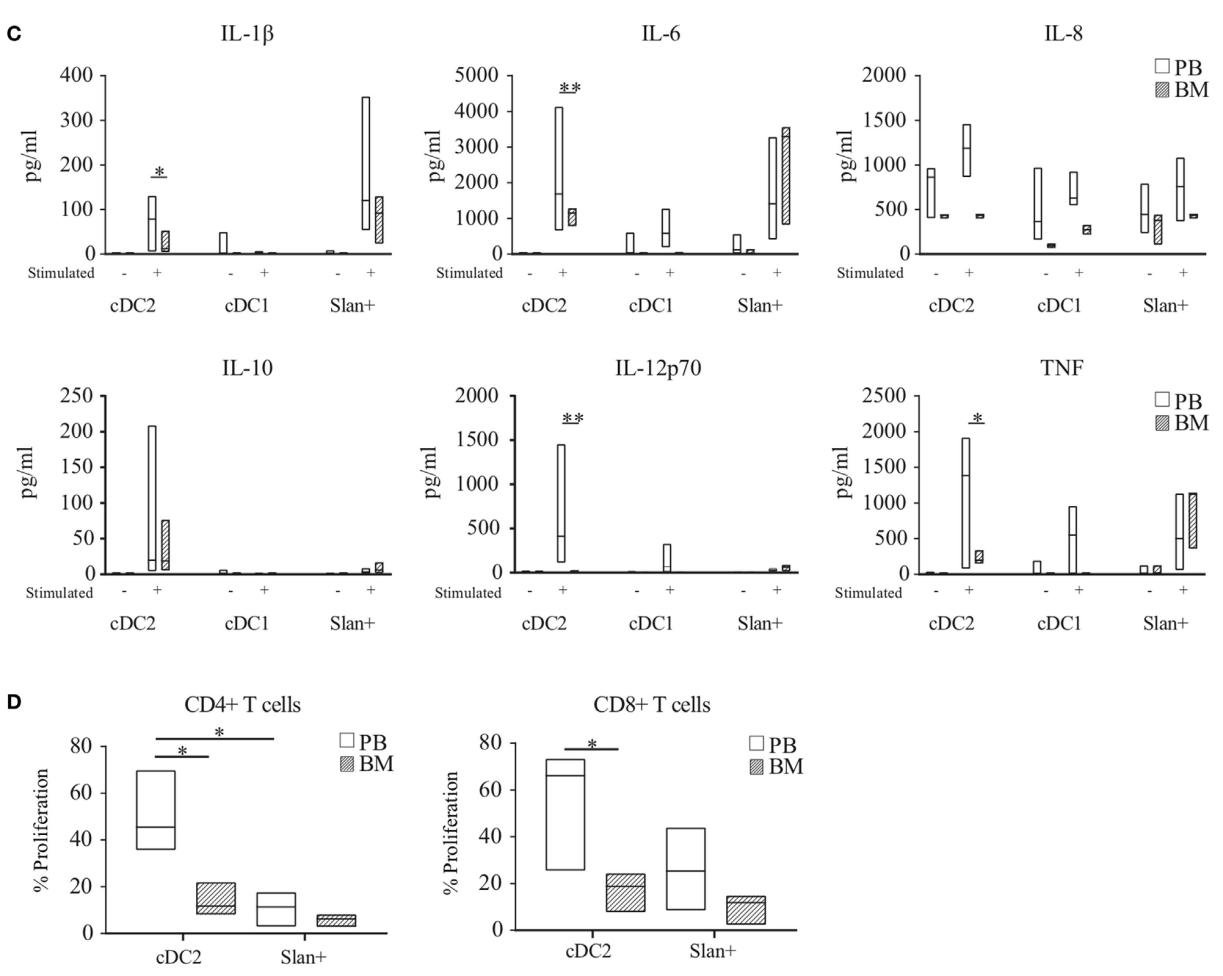
Functional validation of transcriptional findings in myeloid DC and slan+ non-classical monocytes. **(A)** Ki-67 expression in peripheral blood (PB) and bone marrow (BM) subsets. A representative plot of one donor and percentages of positive cells of eight matched samples are shown. A Wilcoxon matched-pairs signed rank test was used to compare the compartments. **(B)** Maturation capacity upon TLR-stimulation. Isolated PB or BM cDC2 and slan+ monocytes were either left unstimulated or were stimulated overnight with LPS + R848. Expression levels of CD80, CD86, and HLA-DR were assessed by flow cytometry. The fold change (FC) in median fluorescence intensity (MFI) values at baseline and after overnight culture (unstimulated or stimulated condition) was calculated. Median FC values for 6–10 PB and 3–4 BM (cDC2) or 8–12 PB and 3–4 BM (slan+) experiments are shown. **(C)** Cytokine secretion assay. Culture supernatants of PB-derived cDC1 (*n* = 3), cDC2, and slan+ (*n* = 6) cells and BM-derived cDC1 (*n* = 2), cDC2, and slan+ (*n* = 4) cells, unstimulated and stimulated (cDC2 and slan+: LPS + R848, cDC1: Poly I:C + R848), were analyzed for the presence of different cytokines by cytometric bead array (CBA, pg, picogram). **(D)** Allogeneic mixed leukocyte reaction (MLR). CFSE-labeled peripheral blood lymphocytes (PBL) were co-cultured with PB or BM-derived cDC2 or slan+ monocytes. The percentage of CFSE-diluted T cells was determined by using flow cytometry. Median values of four different experiments are shown. *p < 0.05, **p < 0.01, and ***p < 0.001.

From the transcriptional profiling as described above it became clear that gene expression in myeloid DC subsets, and to a lesser extent in slan+ non-classical monocytes, was influenced by the compartment in which they reside. For functional validation of these findings, the expression of the Ki-67 protein, an intracellular marker for cell proliferation, was evaluated in a paired analysis of eight matched PB and BM samples. Consistent with our transcriptional analyses, for all subsets the percentage of Ki-67-positive cells was higher in BM than in PB and overall much higher in the DC subsets than in the slan+ non-classical monocytes. This was consistent for all donors (Figure 6A). Furthermore, DC-like functions, such as maturation capacity upon TLR-ligation, were tested for PB- and BM-derived cell subsets. Median fluorescence intensity (MFI) of the co-stimulatory molecules CD80 and CD86, and, of HLA-DR at baseline and after overnight culture in the presence or absence of LPS and R848 (a combination of TLR ligands that has synergistic effects on cytokine production) (30, 39), were compared and fold change values were calculated (Figure 6B). Due to low cell yield, cDC1 could not be tested. The up-regulation of CD80 and CD86 was lower in BM-derived cDC2 as compared to their PB equivalents. In contrast, slan+ non-classical monocytes from BM and PB displayed a similar ability to enhance the expression levels of these molecules.

Next, the cytokine profile of slan+ non-classical monocytes and myeloid DC from the PB and BM compartment was assessed. Cells were stimulated overnight and by using CBA kits different cytokines were analyzed in culture supernatants. For stimulated cDC2 a remarkable difference was found between the PB and BM compartment (Figure 6C). Pro-inflammatory cytokines, in particular the Th-1-polarizing cytokine IL-12p70, were secreted in very low amounts by BM DC as compared to the corresponding subset in PB. A same trend was seen for cDC1 in stimulated conditions. Slan+ non-classical monocytes secreted negligible levels of IL-12p70 in PB and BM. In both compartments, they mainly produced IL-1β, IL-6, IL-8, and TNF, without significant differences between these two sites. Cell survival percentages in these cultures could not be an underlying explanation for differences in cytokine secretion, since cultures showed equal live cell percentages for PB and BM-derived subsets (Figure S3 in Supplementary Material). To test the T cell priming and proliferation induction capacity, stimulated cDC2 and slan+ non-classical monocytes from both compartments were used in an allogeneic MLR. Again, cDC1 could not be used because of low cell numbers. In agreement with the transcriptional results, PB-derived cDC2 showed a superior induction capacity of T cell proliferation in CD4+ T cells as well as in CD8+ T cells compared to the BM-derived cDC2 (Figure 6D; Figure S4 in Supplementary Material). In contrast, but also confirming transcriptional data, slan+ non-classical monocytes did not show a significant difference between the compartments.

## Discussion

Dendritic cell subsets arise in the BM and leave this compartment in a more differentiated, but immature form, in order to supply the peripheral DC pool. The entire human DC development pathway has recently been elucidated and DC progenitors can now be immunophenotypically identified (40). The direct progeny of the circulating DC progenitors (pre-CDC) consists of the well described CD1c+ (cDC2) and CD141+ (cDC1) myeloid DC subsets. These two subsets have been widely studied in a healthy and diseased context, in PB and secondary lymphoid organs such as tonsils, lymph nodes, and spleen (35, 41–44). Their exact developmental state and immunological role in the human BM compartment, however, have not been explored before. Therefore, we carried out a genomic as well as a functional study in which we compared both DC subsets and the slan+ non-classical monocytes from BM to their PB counterparts.

The frequency of different subsets in the BM and PB already showed compartment-related differences. cDC1 were more frequent in the BM compartment than in the PB, whereas slan+ non-classical monocyte levels were clearly higher in PB compared to BM. Initially, slan+ non-classical monocytes were considered to belong to the DC lineage (38, 45), but recent insights firmly place them in the monocyte population (also confirmed in our previous study) (30, 32). The immunophenotypic analysis performed in our current study also supports this reallocation for BM-derived slan+ non-classical monocytes. They showed significantly lower expression levels of HLA-DR as compared to the myeloid DC subsets and similar levels as compared to their PB counterparts and CD14+ classical monocytes. Additional evidence is provided by our transcriptional findings. BM-derived slan+ non-classical monocytes clearly clustered away from the cDC2 and cDC1 and showed the most distinct gene signature with the highest number of DEGs. These differentially expressed transcripts could be translated into pathways that were related to involvement in innate immune responses and a low capacity for DC-like functionalities such as antigen processing and presentation, comparable to PB findings (30). For BM DC subsets, over-expressed genes confirmed their lineage commitment and a consistent subset-specific gene expression profile between PB and BM. However, beside these conserved transcripts, large differences in gene expression were found between the two compartments. In a hierarchical clustering, DC from one compartment clustered closer together than that they did with their equivalent in the other compartment, indicating a location-dependent transcriptional profile. Both cDC2 and cDC1 showed similar enrichment patterns in BM and PB. In BM, a network of cell cycle and differentiation transcripts was found to be over-expressed, whereas in PB, immune-related effector genes were overrepresented. The development of DC is highly dependent on Fms-related tyrosine kinase 3 ligand (46–48). Its receptor, Flt3 (CD135), is expressed in all stages, but highest levels can be found in the most immature forms. From our transcriptional data, both subsets showed over-expression of *FLT3* in the BM compartment, thereby supporting the hypothesis that BM-derived cDC2 and cDC1 are in a more immature and differentiating state than their PB equivalents. Validation of the transcriptional findings by evaluating the expression levels of the proliferation marker Ki-67, further confirmed the FLT3-responsive and proliferative state of BM DC as compared to PB DC. From a functional perspective, a similar conclusion can be drawn. DC from the BM compartment showed a lower activity in DC-like functionalities as compared to matched DC from PB. Upon TLR-triggering, using a known synergistic combination of LPS and R848, BM-derived cDC2 were less able to up-regulate co-stimulatory molecules (in particular CD80), which was reflected in their low capacity for T cell priming. The induction of CD4+ and CD8+ T cell proliferation was substantially lower for BM cDC2 than for PB cDC2. Additionally, the ability of BM DC to secrete pro-inflammatory cytokines was inferior to PB DC. In particular, the production of IL-12p70, a cytokine specifically secreted by highly specialized DC subsets, was nearly absent in BM subsets. For slan+ non-classical monocytes the differences between the two compartments was less evident. Their non-DC-like character was confirmed in both compartments, suggesting minimal effects of the location and a more stable and differentiated state of this cell type.

In conclusion, this study provides the first transcriptional and functional findings for myeloid DC subsets in the human BM. In this compartment, DC harbor more immature and less differentiated features and are not equipped yet to fully exert DC-like functions. These data reveal the BM primarily as a DC developmental location rather than an effector site. Slan+ non-classical monocytes, on the other hand, seem to enter the BM as effectors from the PB in accordance to their more differentiated state and patrolling immune function. In several BM-derived diseases, such as certain hematological malignancies, DC function, and frequency is disturbed potentially causing altered immune responses that may be involved in the pathogenesis of the disease. However, most of these studies focused on PB-derived DC, and only some studied the equivalent forms of DC in the BM compartment (49–57). Since disease development in these disorders probably arises in the BM compartment itself, it is of high interest to study altered immune processes in this compartment rather than only in the PB. Results from this study on healthy BM DC may thus serve as a baseline for further immunological research on aberrant DC function in such disorders.

## Ethics Statement

The study was approved by the local ethical committee. All subjects gave written informed consent in accordance with the Declaration of Helsinki.

## Author Contributions

Contribution: NL-K and KL performed experiments and analyzed data. NL-K, KL, TW, HB, SK, TG, ML, and AL interpreted data. NL-K, KL, TG, ML, and AL designed research. NL-K wrote the paper.

## Conflict of Interest Statement

The authors declare that the research was conducted in the absence of any commercial or financial relationships that could be construed as a potential conflict of interest.
