# Supplementary material for: Human Bone Marrow-Derived Myeloid Dendritic Cells Show an Immature Transcriptional and Functional Profile Compared to Their Peripheral Blood Counterparts and Separate from Slan+ Non-Classical Monocytes
Source: Front Immunol. 2018 Jul 16;9:1619. doi: 10.3389/fimmu.2018.01619 (PMC6055354; doi:10.3389/fimmu.2018.01619)
Supplement: Supplementary file 1 [file data_sheet_1.PDF]

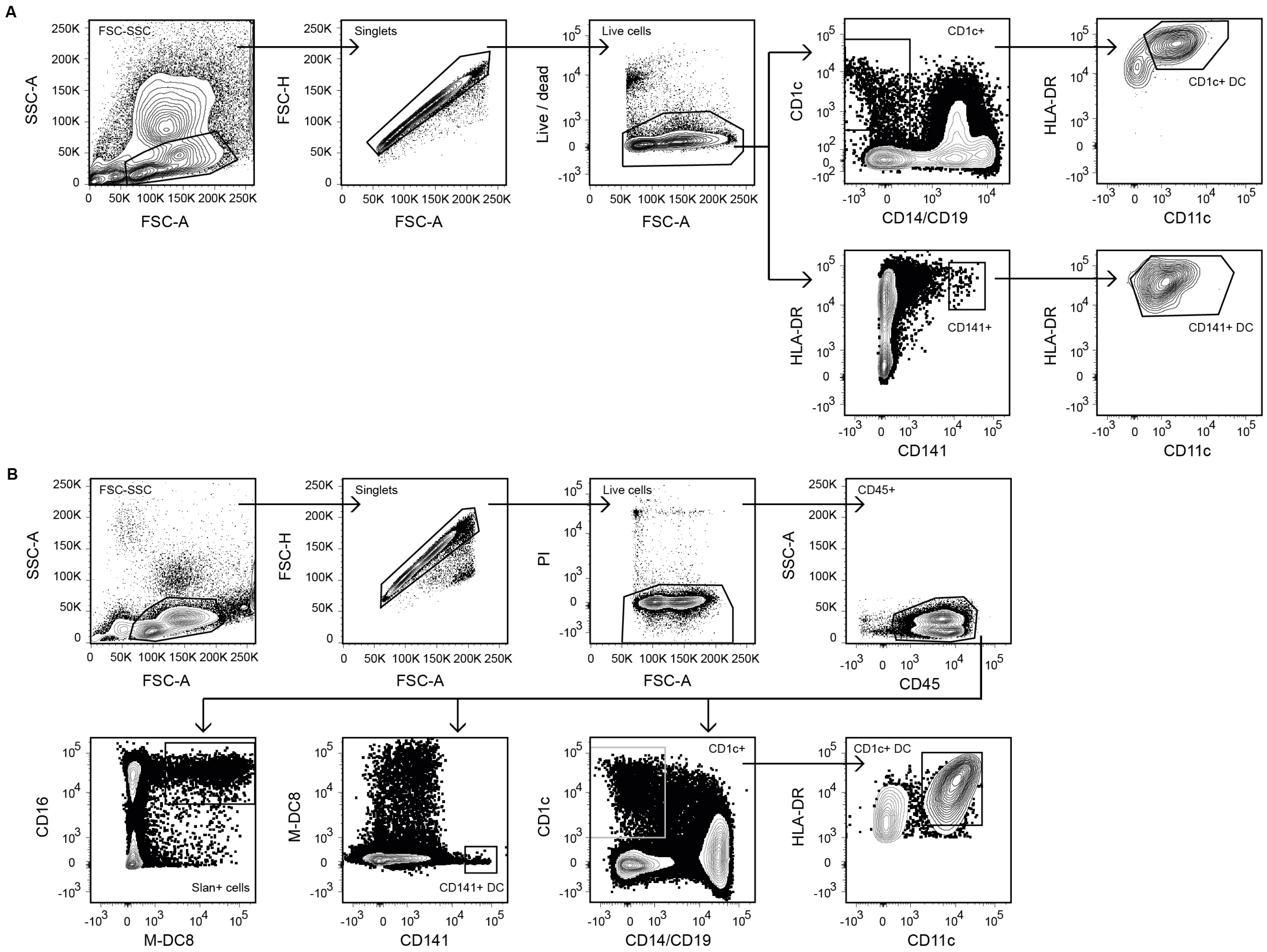

Supplemental figure 1. Sorting strategy of different cell subsets. (A) CD1c+ and CD141+ DC were FACS sorted for functional experiments. After debris and dead cell removal, CD1c+ DC and CD141+ DC were gated. Using HLA-DR and CD11c, pure populations could be sorted. **(B)** For microarray experiments all cell fractions were FACS sorted, including slan+ non-classical monocytes. First, viable cells were gated using propium iodide (PI). Then, different markers were used for subset selection. Slan+ monocytes were gated based on CD16 and M-DC8 expression. CD1c+ DC were obtained from the CD14/CD19 negative cell fraction and further purified by using HLA-DR and CD11c. High expression of CD141 was used for sorting of the CD141+ DC.
