# Supplementary material for: Human Bone Marrow-Derived Myeloid Dendritic Cells Show an Immature Transcriptional and Functional Profile Compared to Their Peripheral Blood Counterparts and Separate from Slan+ Non-Classical Monocytes
Source: Front Immunol. 2018 Jul 16;9:1619. doi: 10.3389/fimmu.2018.01619 (PMC6055354; doi:10.3389/fimmu.2018.01619)
Supplement: Supplementary file 2 [file data_sheet_2.PDF]

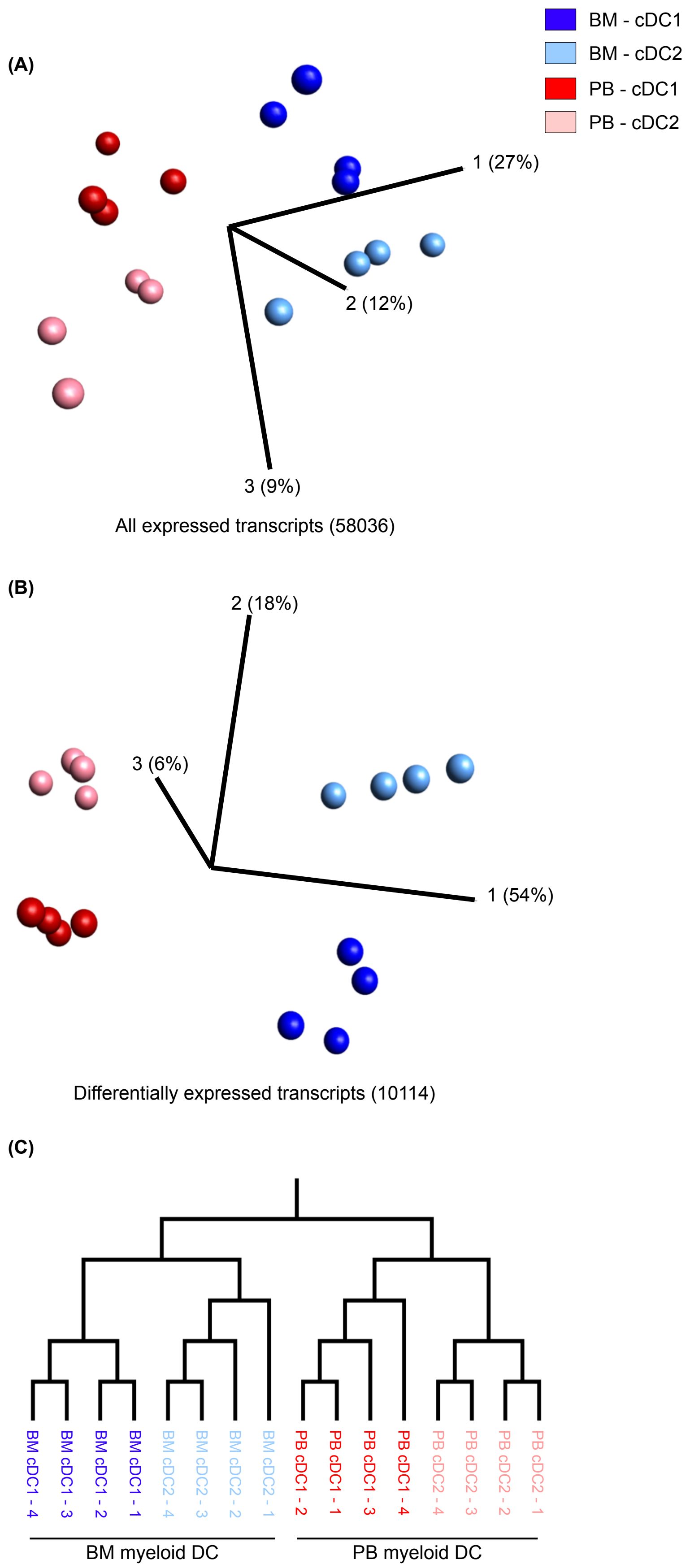

Supplemental figure 2. PCA and hierarchical clustering for DC subsets only. Relationship between cDC1 and cDC2 in the peripheral blood and bone marrow. **(A)** Principal component analysis (PCA) of all expressed transcripts and **(B)** for differentially expressed transcripts. Percentages shown reflect the degree of total variation within each component. **(C)** Hierarchical clustering, based on the top 10,000 differentially expressed genes, of replicate samples.
