# Supplementary material for: Human Bone Marrow-Derived Myeloid Dendritic Cells Show an Immature Transcriptional and Functional Profile Compared to Their Peripheral Blood Counterparts and Separate from Slan+ Non-Classical Monocytes
Source: Front Immunol. 2018 Jul 16;9:1619. doi: 10.3389/fimmu.2018.01619 (PMC6055354; doi:10.3389/fimmu.2018.01619)
Supplement: Supplementary file 3 [file data_sheet_3.PDF]

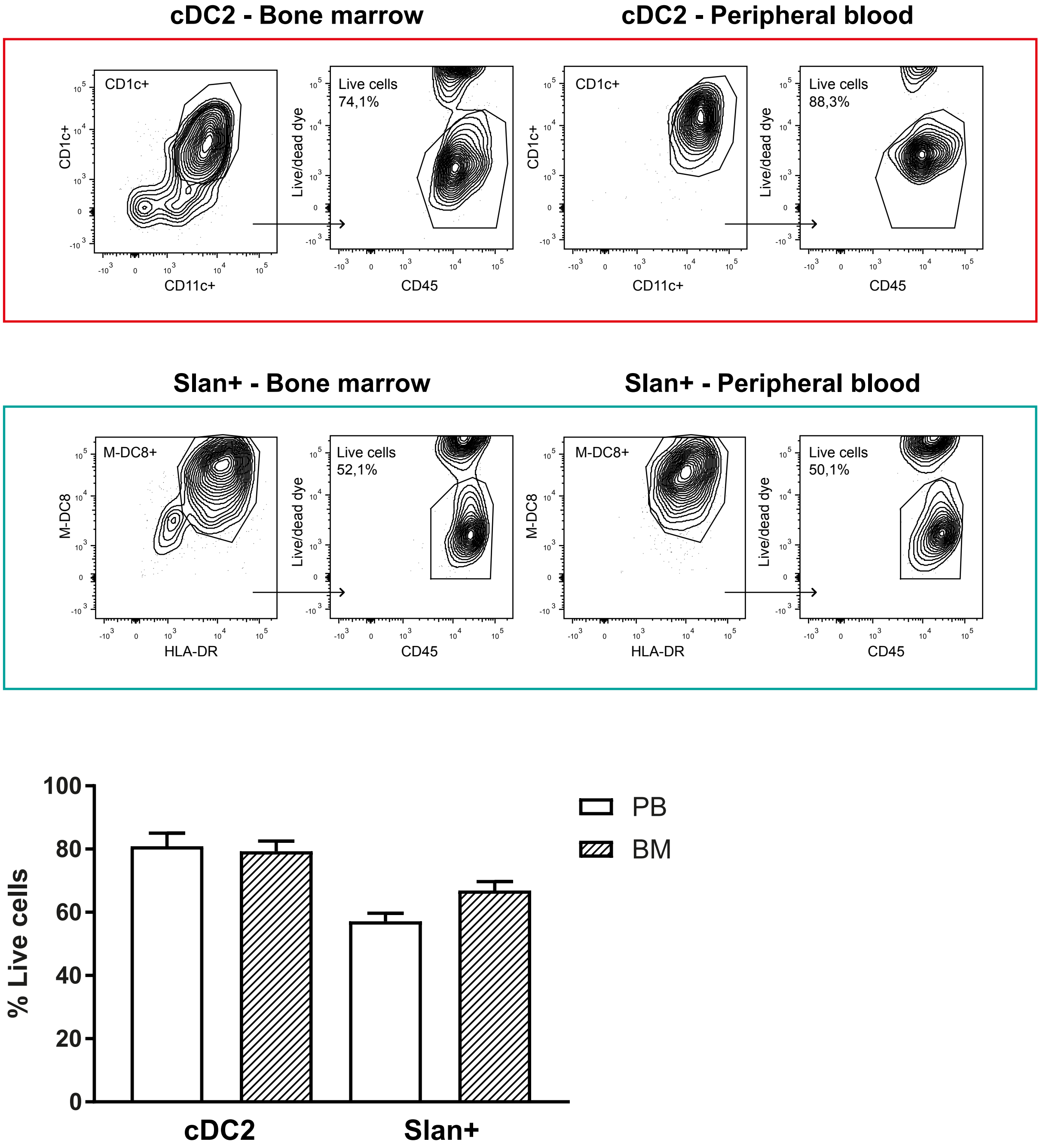

Supplemental figure 3. Cell survival in cultures. Isolated subsets were cultured overnight and were stimulated with a combination of TLR-agonist (LPS+R848). Next day, they were harvested and stained with different maturation markers. Culture supernatants were used for cytokine analysis. Cell survival in these cultures were not different between bone marrow and peripheral blood derived cDC2 (in red) or Slan+ cells (in green). Plots of a representative experiment are shown and a bar graph of all experiments is displayed.
