# Supplementary material for: Human Bone Marrow-Derived Myeloid Dendritic Cells Show an Immature Transcriptional and Functional Profile Compared to Their Peripheral Blood Counterparts and Separate from Slan+ Non-Classical Monocytes
Source: Front Immunol. 2018 Jul 16;9:1619. doi: 10.3389/fimmu.2018.01619 (PMC6055354; doi:10.3389/fimmu.2018.01619)
Supplement: Supplementary file 4 [file data_sheet_4.PDF]

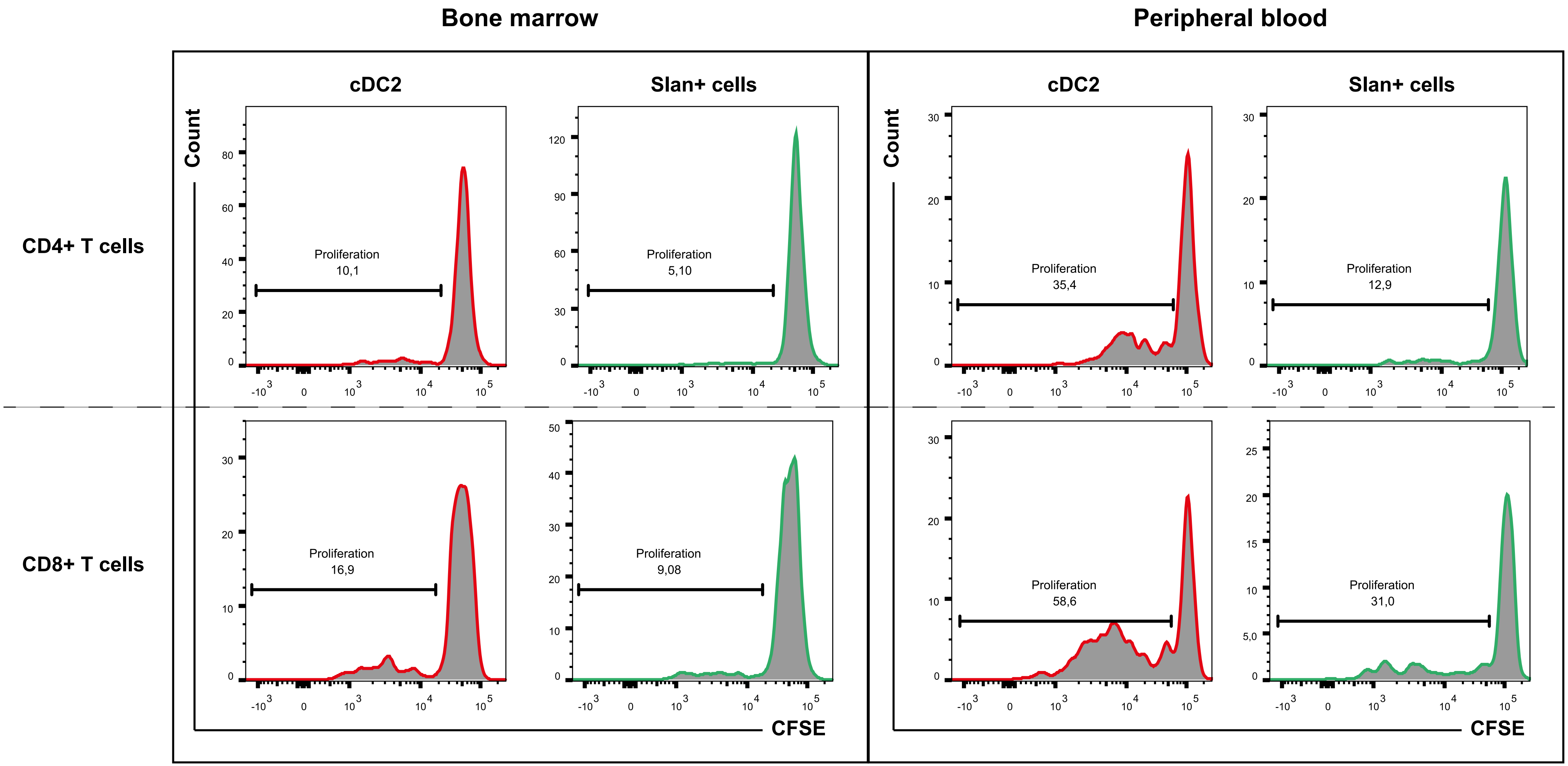


**Supplemental figure 4. Mixed leukocyte reaction (MLR).** CFSE-labeled peripheral blood lymfocytes (cell fraction with depletion of CD14+ cells) were co-cultured with isolated and stimulated cDC2 (in red) or slan+ cells (in green) either derived from bone marrow or from peripheral blood samples. After 5 days, cells were harvested and stained with a live/dead dye and anti-CD3, anti-CD4 and anti-CD8 antibodies. CFSE dilution over daughter cells was analyzed using flow cytometry. Representative histograms of the experiment are shown. The percentage proliferation is displayed.
